# IFNβ Protects Neurons from Damage in a Murine Model of HIV-1 Associated Brain Injury

**DOI:** 10.1038/srep46514

**Published:** 2017-04-20

**Authors:** Victoria E. Thaney, Alan M. O’Neill, Melanie M. Hoefer, Ricky Maung, Ana B. Sanchez, Marcus Kaul

**Affiliations:** 1 Infectious and Inflammatory Disease Center, Sanford Burnham Prebys Medical Discovery Institute, 10901 North Torrey Pines Road, La Jolla, CA 92037, USA; 2 Graduate School of Biomedical Sciences, Sanford-Burnham Prebys Medical Discovery Institute, 10901 North Torrey Pines Road, La Jolla, CA 92037, USA; 3 Department of Psychiatry, University of California, San Diego, 9500 Gilman Drive, San Diego, CA 92093, USA

## Abstract

Infection with human immunodeficiency virus-1 (HIV-1) causes brain injury. Type I interferons (IFNα/β) are critical mediators of any anti-viral immune response and IFNβ has been implicated in the temporary control of lentiviral infection in the brain. Here we show that transgenic mice expressing HIV-1 envelope glycoprotein 120 in their central nervous system (HIVgp120tg) mount a transient IFNβ response and provide evidence that IFNβ confers neuronal protection against HIVgp120 toxicity. In cerebrocortical cell cultures, neuroprotection by IFNβ against gp120 toxicity is dependent on IFNα receptor 1 (IFNAR1) and the β-chemokine CCL4, as IFNAR1 deficiency and neutralizing antibodies against CCL4, respectively, abolish the neuroprotective effects. We find *in vivo* that IFNβ mRNA is significantly increased in HIVgp120tg brains at 1.5, but not 3 or 6 months of age. However, a four-week intranasal IFNβ treatment of HIVgp120tg mice starting at 3.5 months of age increases expression of CCL4 and concomitantly protects neuronal dendrites and pre-synaptic terminals in cortex and hippocampus from gp120-induced damage. Moreover, *in vivo* and *in vitro* data suggests astrocytes are a major source of IFNβ-induced CCL4. Altogether, our results suggest exogenous IFNβ as a neuroprotective factor that has potential to ameliorate *in vivo* HIVgp120-induced brain injury.

Infection with HIV-1 often causes cognitive problems, which are referred to as HIV-associated neurocognitive disorders (HAND)^1^. HIV-associated dementia (HAD) is the most severe manifestation^1^,^2^,^3^, and remains an independent risk factor for death due to AIDS despite combination anti-retroviral therapy (cART)^4^. The pathogenesis of HAND is still incompletely understood and there is currently no specific treatment available.

The neuropathology associated with HIV infection of the brain is characterized by astrocytosis, myelin pallor, infiltration of macrophages (MΦ), increased numbers of resident microglia and multinucleated giant cells^5^,^6^. The pathological features most closely associated with the clinical signs of HAND/HAD comprise an increased number of microglia and MΦ, evidence of excitotoxins, decreased synaptic and dendritic density, and selective neuronal loss^7^,^8^,^9^. The affected brain regions include the frontal cortex, hippocampus, substantia nigra, putamen, basal ganglia and cerebellum (reviewed in refs 9, 10, 11, 12).

One model for brain damage seen in AIDS patients are transgenic (tg) mice expressing the gp120 of the HIV-1 isolate LAV under the control of a modified GFAP promotor in astrocytes in their central nervous system (CNS)^13^. HIV-1_LAV_ is a CXCR4-utilizing viral strain that infects MΦ and lymphocytes^14^, and HIVgp120tg mice manifest several neuropathological features observed in AIDS brains, such as decreased synaptic and dendritic density, increased numbers of activated microglia and pronounced astrocytosis^13^. HIVgp120tg mice also develop significant behavioral changes, such as impaired spatial learning and memory at 8 months^15^ and reduced swimming velocity at 12 months of age^16^. Moreover, our recent analysis of differential CNS gene expression comparing HIVgp120tg and non-tg, wild-type (WT) control mice revealed a significant overlap for differentially expressed genes between brains of HIVgp120tg mice and human HIV and HIV encephalitis (HIVE) patients^15^,^17^.

HIVgp120tg mouse brains also displayed a gene expression pattern consistent with an endogenous IFN response^15^,^18^. The innate immune system responds to HIV-1 infection with the production of interferons (IFNs)^19^,^20^,^21^. Type I IFNs (IFNα and IFNβ) exert their biological functions through interaction with the type I interferon (α/β) receptors (IFNAR1 and −2) while the type II IFN (IFNγ) binds to a distinct IFNγ receptor (IFNGR)^22^. IFNs regulate many components in an immune response against infections and promote - in an autocrine or paracrine fashion - an anti-viral state that is characterized by the expression of distinct IFN-stimulated genes (ISG)^22^,^23^. Those include among others IFN regulatory factor (IRF)-1 and oligoadenylate synthetase-1 (OAS1), which interfere with viral replication^23^,^24^ and are up-regulated in brains of HIVgp120tg mice^15^,^18^.

The lasting expression of IFNα in the HIV-1 exposed CNS has been linked to cognitive impairment and inflammatory neuropathology^25^,^26^. In contrast, IFNβ has been implicated in the control of HIV and SIV infection in the brain^27^,^28^. Exposure to IFNβ induces in lymphocytes, MΦ and microglia the expression and secretion of the β-chemokines CCL3, −4 and −5. These natural ligands of the HIV co-receptor CCR5 have been shown to suppress HIV-1 infection and disease progression^29^,^30^, and we have implicated CCL4 and CCL5 in neuroprotection^31^,^32^. IFNβ has pronounced anti-inflammatory effects as it inhibits the induction of MHC antigens and down-regulates expression of Th1 cytokines (e.g. IL-2, IL-12, IFNγ) and iNOS triggered by IFNγ in glial cells^33^,^34^,^35^. Moreover, IFNβ is currently used to treat multiple sclerosis (MS)^36^,^37^. Thus, it seemed reasonable to further investigate whether IFNβ as part of the innate anti-viral immune response can provide a beneficial effect against the neurotoxicity of HIV-1.

Here we report the occurrence of a transient endogenous IFNβ-driven type I IFN response in the brains of HIVgp120tg mice at 1.5 months of age and provide evidence for a neuroprotective function of exogenously supplied IFNβ. *In vitro* experiments showed that IFNβ protected cerebrocortical neurons against gp120 toxicity while requiring the presence of IFNAR1 and CCL4. Immunohistological analysis revealed that a four-week, once-a-week intranasal IFNβ treatment regimen prevented neuronal damage *in vivo* in 3–4 month old HIVgp120tg mice. Both *in vitro* and *in vivo* experiments suggested that astrocytes were a major source of IFNβ-induced CCL4. Altogether, our findings suggested IFNβ is a neuroprotective factor with potential to ameliorate *in vivo* brain injury induced by neurotoxic components of HIV-1.

## Results

### Brains of HIVgp120tg mice express mRNA of IFNβ and IFN-stimulated genes

We previously observed in a CNS gene expression study that HIVgp120tg mice mount an IFN response^15^. This recent study investigated two founder lines of HIVgp120tg mice (line 1 (L1) and 2 (L2)). Common to both HIVgp120tg mouse lines is the differential regulation of 1,195 genes in gp120-expressing brains in comparison to non-tg littermate controls. Here we evaluated the 1,195 differentially expressed genes for upstream regulators employing the commercially available software Ingenuity Pathway Analysis (IPA)^15^. Using the core analysis function of IPA we identified IFNβ as a major upstream regulator of the differential gene pattern associated with gp120 expression (p-value overlap 1.01 × 10^−12^).

**Figure 1:**
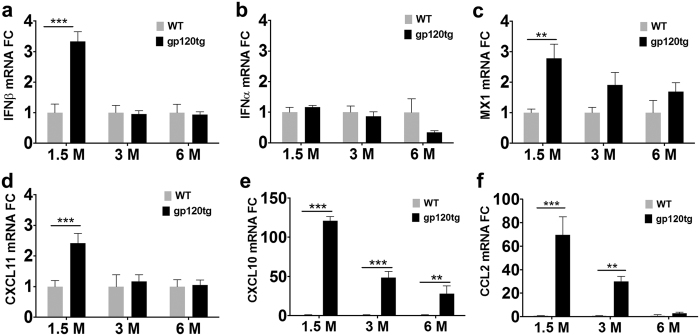
Transient IFNβ expression is accompanied by up-regulation of interferon-stimulated genes in HIVgp120tg mouse brains. Total brain RNA was extracted from age-matched HIVgp120tg and wild type control, male animals and qRT-PCR analysis was used to assess differential regulation of the indicated genes between the age-groups: (**a**) IFNβ; (**b**) IFNα; (**c**) MX1; (**d**) CXCL11; (**e**) CXCL10; (**f**) CCL2. GAPDH was used as internal control, and representative results are shown as fold-change (FC) differences compared to the WT control within the age group. Data are shown as mean ± s.e.m. (***P ≤ 0.001, **P ≤ 0.01 by ANOVA with Fisher’s PLSD post hoc test). *n* = 3–4 animals per group/genotype.

As a follow up, we investigated the expression of IFNβ and -α, and a number of ISGs, including MX1, CXCL11, CXCL10, and CCL2, in the brains of HIVgp120tg mice and WT-littermate control (L2) using quantitative reverse transcription polymerase chain reaction (qRT-PCR). L2 mice were chosen because they showed a slightly more pronounced neuropathology than L1 animals in our previous study^15^. RNA was isolated from male animals of 1.5, 3 and 6 months of age and qRT-PCR performed as described in the Methods section. Low level baseline expression of IFNα/β in the CNS was observed as expected based on recent reports by others^38^,^39^ (Fig. 1a,b). However, HIVgp120tg brains displayed a significant increase in IFNβ mRNA levels at 1.5, but not at 3 or 6 months of age relative to WT controls (Fig. 1a). In contrast, IFNα was not significantly elevated in HIVgp120tg brains compared to WT controls (Fig. 1b). The roughly 2-fold increase in IFNβ gene expression was accompanied by an up-regulation of ISGs, such as MX1, CXCL11, CXCL10 and CCL2, which all showed the highest levels at 1.5 months, followed by a decline in animals of 3 to 6 months of age (Fig. 1c–f). The expression kinetics were similar for IFNβ and CXCL11 with transcript levels back at baseline at 3 months. In contrast, MX1, CXCL10 and CCL2 showed a more gradual decrease from 1.5 to 6 months. At 1.5 months, mRNAs of MX1 and CXCL11 displayed a modest but significant 1.8 and 1.4 fold increases, but the most pronounced changes in mRNA levels occurred for CCL2 and CXCL10, which were 69 and 120 fold up-regulated, respectively, compared to WT. These results were in line with the findings of the bioinformatics and earlier microarray analysis and demonstrated that HIVgp120tg mice mounted an endogenous IFNβ response.

### IFNβ protects cerebrocortical neurons against toxicity of HIV-1 gp120

**Figure 2:**
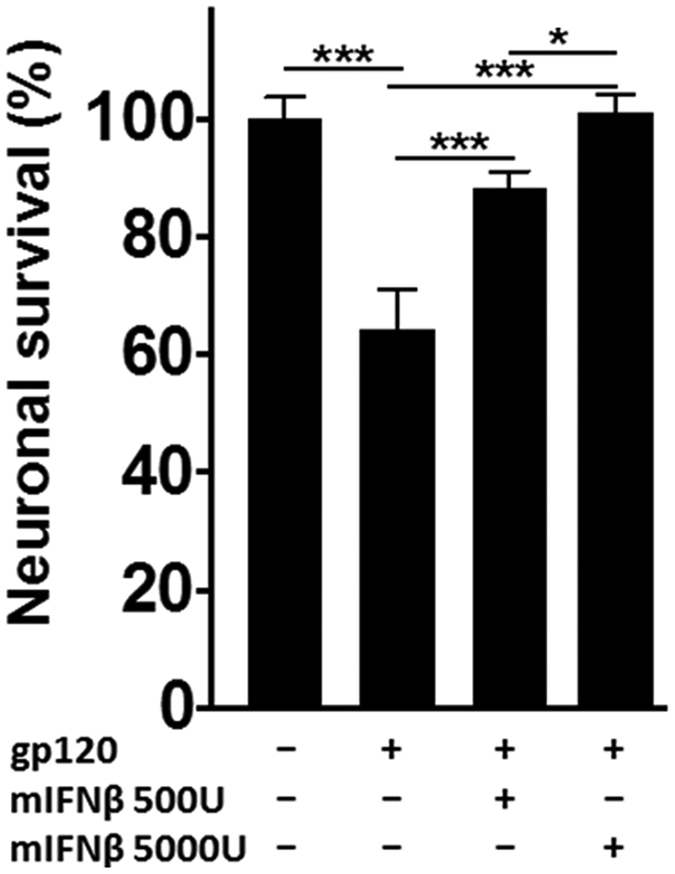
IFNβ mediates neuroprotection against HIV gp120-induced injury. Rat cerebrocortical cultures were treated with gp120 from HIV-1 strain BaL (200 pM) for 24 h in the presence or absence of different IFNβ concentrations (500 or 5,000 U/ml) or BSA/PBS (0.001% final concentration) as vehicle control. Following the incubation the cells were fixed and permeabilized, and neurons were immunolabeled for neuronal MAP-2 and NeuN while nuclear DNA was stained with H33342. Neuronal survival was assessed using fluorescence microscopy and cell counting as described in Methods. Values are mean ± s.e.m.; n = 2 independent experiments with 4–7 replicates and an average of 5,300 cells counted per condition; ***p < 0.001, **p < 0.01, *p < 0.05 by ANOVA with Fisher’s PLSD post hoc test.

Cerebrocortical cell cultures contain neurons, astrocytes, and microglia that express both CXCR4 and CCR5 co-receptors and provide an established model system to study the neurotoxic action of HIVgp120^32^. In order to determine if IFNβ can protect neurons against toxicity induced by the viral envelope protein, we exposed cerebrocortical cells derived from rat to recombinant gp120 of the macrophage-tropic HIV-1 strain BaL in the presence or absence of 500 or 5,000 U/ml of recombinant murine IFNβ (mIFNβ). Following 24 h of incubation the cells were fixed with 4% paraformaldehyde (PFA) and neuronal survival was assessed using fluorescence microscopy as described in the Method section. Counts of MAP-2 and NeuN double-positive neurons in the total cell number were used to compare the experimental groups. Cells exposed to BSA/PBS vehicle served as control and the number of neurons in this experimental condition was defined as 100% survival. Exposure to gp120 decreased the percentage (±s.e.m.) of surviving neurons significantly to 64.2 ± 6.9% (Fig. 2). However, when the cerebrocortical cells were exposed to gp120 in the presence of 500 U/ml of mIFNβ, the percentage of surviving neurons was significantly greater (88.3 ± 2.8%) compared to gp120 alone. 5,000 U/ml mIFNβ completely abrogated gp120 toxicity with the percentage of surviving neurons (101.1 ± 3.1%) being indistinguishable from vehicle control. Thus, IFNβ counteracted gp120-induced neurotoxicity in a dose-dependent manner. Of note, neuronal survival remained unaffected upon exposure to IFNβ at 5,000 U/ml in the absence of gp120 (Supplementary Fig. S1a).

### IFNβ induces ISGs and β-chemokines in cerebrocortical cells with distinct kinetics

In order to further characterize the neuroprotective effect of IFNβ, we next investigated the potential induction of the ISG and natural CCR5 ligands CCL3, CCL4 and CCL5 because they suppress HIV-1 infection^29^,^30^, and CCL4 and CCL5 may protect neurons against HIVgp120 toxicity^32^. CXCL10 was chosen because it has been implicated in neuronal toxicity^40^ and was elevated in the CNS of patients suffering with HIVE^41^ and HIVgp120tg mice (Fig. 1e).

**Figure 3:**
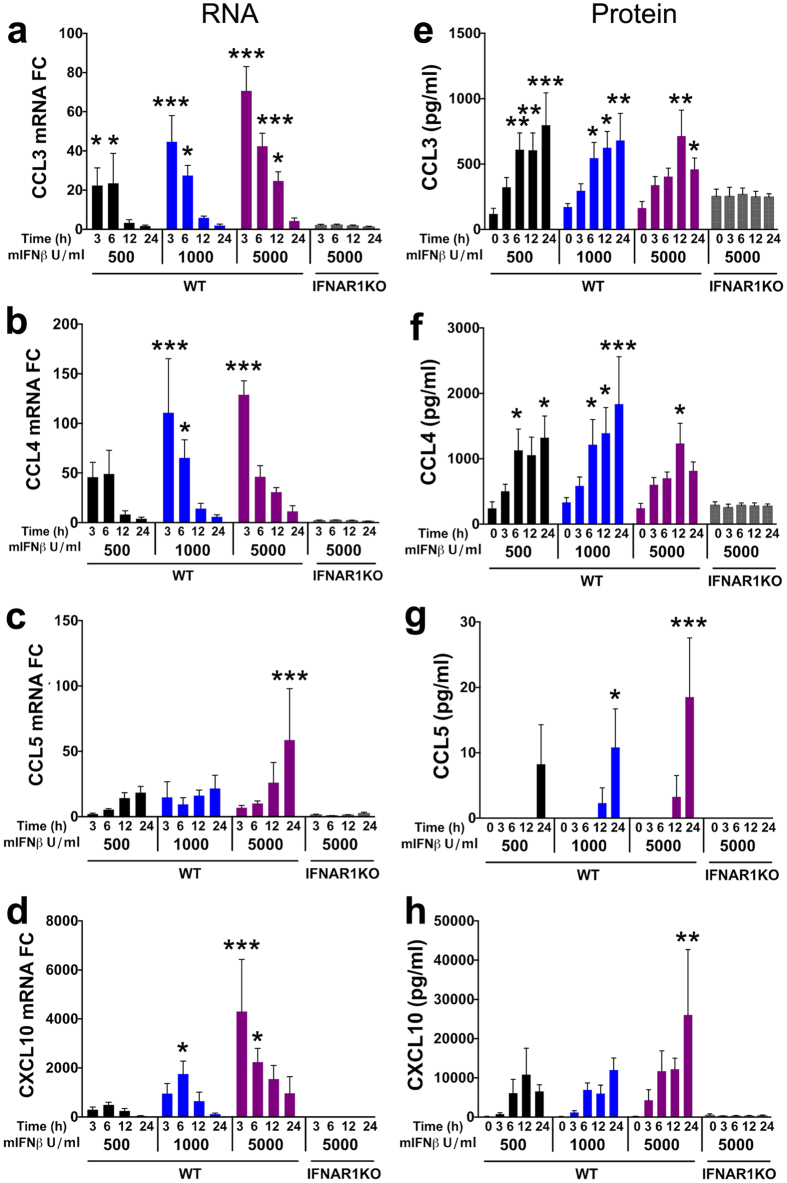
IFNβ stimulates expression of interferon-stimulated genes (ISG) in mixed neuronal-glial cerebrocortical cell cultures. Mixed neuronal-glial cerebrocortical cultures from WT or IFNAR1KO mice were incubated with IFNβ in increasing doses (from 500 to 5,000 U/ml) or BSA/PBS vehicle control for 0, 3, 6, 12 and 24 h. Total RNA was extracted from cell lysates, analyzed by qRT-PCR and normalized to GAPDH expression levels. (**a–d**) RNA expression is shown as fold change (FC) in relation to vehicle treated controls which were defined as baseline activity. (**e–h**) Time course for protein expression measured in cell-free supernatants for CCL3, CCL4, CCL5 and CXCL10 using a commercially available multiplex assay as described in Methods. Baseline protein expression in vehicle treated cell cultures is represented as 0 h time point. Values are mean ± s.e.m.; n = 3–5 independent experiments per ISG; ***p < 0.001, **p < 0.01, *p < 0.05 by ANOVA with Fisher’s PLSD post hoc test. For clarity, the significance is only indicated for differences between treatments and baseline within each experimental group.

In order to examine ISG induction by recombinant IFNβ, we chose cerebrocortical cell cultures derived from mice, because a genetic knockout of IFNAR1 (IFNAR1KO) has been generated in this model system^42^ and exposure of cerebrocortical cultures from IFNAR1 WT mice to recombinant mIFNβ at 5,000 U/ml, the highest concentration used in the present study, did not affect neuronal survival (Supplementary Fig. S1b). We analyzed kinetics of mRNA expression and protein concentration following 3, 6, 12 and 24 h treatment with different concentrations of recombinant mIFNβ, in mixed neuronal-glial cerebrocortical cells of both WT and IFNAR1KO. Lipopolysaccharide (LPS) was used as a pro-inflammatory control that can activate chemokine expression independently of IFNβ via a pathway involving toll-like receptor (TLR)-4^43^. Treatment of WT cerebrocortical cells with 500, 1,000 or 5,000 U/ml mIFNβ increased baseline mRNA expression of all four chemokines, whereas no effect was detected in IFNAR1KO cells (Fig. 3a–d). As expected, the greatest mRNA induction in WT cells correlated with the highest IFNβ concentration. When examining the temporal induction of each ISG, we found that the mRNA levels of CCL3, CCL4 and CXCL10 were maximal at 3–6 h and then decreased steadily until 24 h (Fig. 3a,b,d). In contrast, expression of CCL5 increased steadily over time, with the highest fold-induction occurring at 24 h (Fig. 3c).

To assess how mRNA kinetics translated into protein, the chemokines were measured in culture supernatants of WT and IFNAR1KO cerebrocortical cells using multiplex assays as described in the Method section. In WT and IFNAR1KO cerebrocortical culture supernatants CCL3, −4 and CXCL10 were abundant at baseline (0 h exposure), but CCL5 was not detected (Fig. 3e–h). Maximal release of CCL3, CCL4 and CXCL10 occurred around 12–24 h post-treatment with mIFNβ (Fig. 3e,f,h) but the protein concentrations of CCL3 and CCL4 in culture supernatants were similar after exposure to 500, 1000 and 5,000 U/ml of mIFNβ. In contrast, CCL5 was mostly below the detection limit of the assay (<3.2 pg/ml) and became detectable only at 12–24 h (Fig. 3g). This pattern followed the mRNA kinetics induced by mIFNβ (Fig. 3c).

However, induction of CCL3, CCL4, CCL5 and CXCL10 was not just dependent on IFNβ exposure, as the expression of all four chemokines was activated by LPS in IFNAR1KO cerebrocortical cells (Supplementary Fig. S2). IFNAR1KO cerebrocortical cells showed higher induction of RNA levels in response to LPS compared to WT cells for all analyzed cytokines with the exception of CXCL10.

In contrast to IFNβ, incubation of cerebrocortical cultures with HIVgp120 at concentrations that cause neurotoxicity (200 pM) did not induce any additional amounts of IFNβ or any of the above mentioned ISG beyond the baseline (Supplementary Fig. S3a).

### Neuroprotection by IFNβ against HIVgp120 toxicity requires IFNAR1 and CCL4

**Figure 4:**
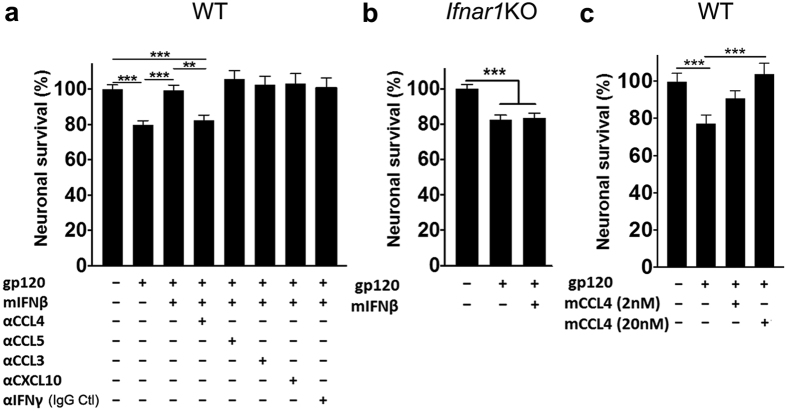
Neuroprotection by IFNβ against HIVgp120 toxicity requires IFNAR1 and CCL4. (**a**) Mixed neuronal-glial cerebrocortical cultures from WT mice were simultaneously exposed for 3 days to HIV gp120_BaL_ (200 pM) and mouse IFNβ (5,000 U/ml) in the presence and absence of neutralizing antibodies against CCL3, CCL4, CCL5, IFNγ or CXCL10. IFNγ antibody was used as control for neutralizing antibodies since this protein was undetectable in cerebrocortical cell cultures. (**b**) Mouse cerebrocortical cultures from IFNAR1KO mice were stimulated with gp120_BaL_ for 24 h the presence or absence of mouse IFNβ (5,000 U/ml) or BSA/PBS control. (**c**) Cerebrocortical cell cultures from WT mice were simultaneously exposed for 3 days to HIV gp120_BaL_ in the presence and absence of murine CCL4 (2 or 20 nM). Neuronal survival was assessed by immunofluorescence microscopy and counting of MAP-2/NeuN double-positive neurons. Values are mean ± s.e.m.; n = 3–5 independent experiments with 3–7 replicates and an average of 9,000 (IFNAR1KO) or 5,700 (WT) cells counted per condition; **p < 0.01, ***p < 0.001 by ANOVA with Fisher’s PLSD post hoc test.

Exposure of WT mouse cerebrocortical cell cultures to HIVgp120_BaL_ (200 pM) for 72 h resulted in a significant decrease in neuronal survival, which was abrogated in the presence of 5,000 U/ml of mIFNβ (Fig. 4a), thus recapitulating our findings in neuro-glial cell cultures from rat (Fig. 2).

IFNβ induced in cerebrocortical cells CCL4 and −5, which we found earlier to be neuroprotective^32^, as well as CCL3 and CXCL10. The latter has been implicated by others in HIV neurotoxicity^44^. Therefore, we investigated the potential contribution of those chemokines in IFNβ-mediated neuroprotection. In order to specifically and quantitatively target the proteins, we employed specific neutralizing antibodies (Abs) against CCL3, CCL4, CCL5 and CXCL10. Mouse cerebrocortical cells were simultaneously incubated for 72 h with gp120 of HIV-1_BaL_ and 5,000 U/ml mIFNβ in the presence and absence of one of the neutralizing Abs (Fig. 4a). Neuronal survival was analyzed by immunofluorescence microscopy and cell counting as described beforehand. An Ab against IFNγ was used as a control IgG, because the type II IFN was not detectable in supernatants of cerebrocortical cells after treatment with IFNβ or gp120 (Supplementary Fig. S3b). Introduction of neutralizing Abs against CCL4, but not CCL5, CCL3, CXCL10 or IFNγ, completely abrogated the neuroprotective effect of IFNβ (Fig. 4a). This result suggested that induction of CCL4 is a crucial component of neuroprotection by IFNβ. Of note, neuronal survival was indistinguishable from vehicle control in mouse cerebrocortical cultures exposed only to IFNβ in the presence and absence of neutralizing Abs (Supplementary Fig. S1b).

On the other hand, IFNβ failed to abrogate gp120-mediated toxicity in cerebrocortical cells from IFNAR1KO mice, confirming the specific requirement of IFNβ signaling through IFNAR1 receptor for neuroprotection (Fig. 4b).

However, incubation of WT mouse cerebrocortical cells with HIVgp120 for 72 h in the presence of recombinant murine CCL4 (mCCL4) at 2 or 20 nM prevented any significant neurotoxic effect (Fig. 4c). In contrast, cells exposed to HIVgp120 in the absence of the chemokine displayed a significant loss of neurons. This finding further supported a critical role of CCL4 in neuroprotection by IFNβ.

### Intranasally administered IFNβ prevents neuronal damage in HIVgp120tg mice

**Figure 5:**
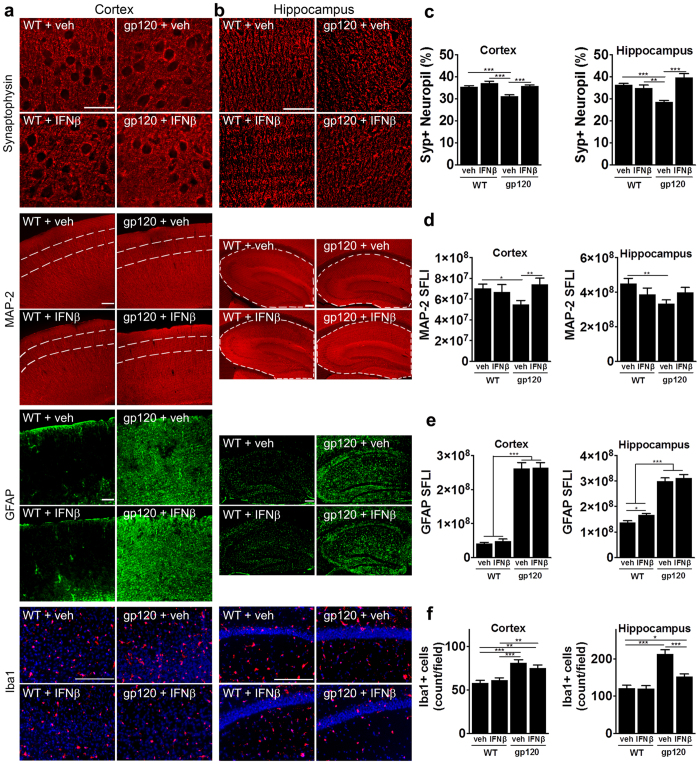
Intranasally administered IFNβ prevents neuronal damage in HIVgp120tg mice. Three to four month-old HIVgp120tg and WT littermate male mice received intranasal recombinant murine IFNβ (50,000 U/25 g bodyweight) or vehicle once a week for four weeks and afterwards, the brains were assessed for neuronal damage and glial activation. Representative images of frontal cerebral cortex (**a**) and hippocampus (**b**) immunolabeled for neuronal synaptophysin (SYP; cortex layer III, hippocampus CA1) deconvolution microscopy; scale bar, 40 μm) and MAP-2 (cortex layer III (area between dashed lines) and hippocampus) fluorescence microscopy; scale bar, 100 μm), astrocytic GFAP (scale bar, 100 μm) and microglial Iba1 (red, DNA in blue, scale bar, 100 μm). (**c–f**) Quantification of microscopy data obtained in frontal cortex and hippocampus of sagittal brain sections of four to five month-old HIVgp120tg mice and controls treated with IFNβ or vehicle: (**c**) SYP in percent positive neuropil, (**d**) MAP-2 immunoreactivity as sum of fluorescence intensity (SFLI; arbitrary units); (**e**) fluorescence signal for astrocytic GFAP; (**f**) quantification of Iba1^+^ microglia (counts/microscopic field). Values are mean ± s.e.m.; ***p < 0.001, **p < 0.01, *p < 0.05; ANOVA and Fisher’s PLSD post hoc test; n = 4–5 animals per group/genotype.

Endogenous IFNβ expression was elevated in HIVgp120tg brains at 1.5 months (Fig. 1a) and *in vitro* IFNβ abrogated neurotoxicity of HIVgp120 (Fig. 2). Therefore, we wondered if delivery of exogenous, recombinant mIFNβ into the brain of HIVgp120tg mice can provide neuroprotection. In order to achieve delivery of IFNβ to the brain and to avoid potential negative side effects in the periphery, we used an intranasal route for CNS delivery as we have previously shown for erythropoietin (EPO) and insulin-like growth factor-I (IGF-1)^45^. Moreover, others have recently shown that intranasal administration of IFNβ is a non-invasive method to deliver the cytokine to the central nervous system in rats and non-human primates^46^,^47^. Here we carried out a 4-week, once a week intranasal treatment with recombinant mIFNβ (50,000 IU/25 g body weight) or vehicle as control on 3–4 month-old HIVgp120tg mice and WT littermates as detailed in the Method section. The age was chosen based on the previous report that HIVgp120tg mice display detectable neuropathological changes in the frontal cortex and hippocampus by 3 months^13^, at which time we found that endogenous IFNβ expression had returned to baseline levels (Fig. 1a). After the last treatment the mice were killed by overdose anesthesia, brain tissue was harvested and CNS damage was assessed by immunohistological analysis. Sagittal brain sections were immune-labeled for pre-synaptic terminals and neuronal dendrites and cell bodies using markers for synaptophysin (SYP) and microtubule-associated protein 2 (MAP-2), respectively. Analysis of frontal cortex (layer III) and the hippocampus (CA1) using deconvolution microscopy showed a significant loss of SYP-positive neuropil (presynaptic terminals) in HIVgp120tg mice that had received vehicle, but not those that were treated with IFNβ, compared to vehicle and IFNβ-exposed WT controls (Fig. 5a–c). Consistent with this finding, quantitative analysis of the immunofluorescence signal for MAP-2 expressed as sum of fluorescent intensities, (SFLI) showed significant loss of this neuronal marker in cortex and hippocampus only in HIVgp120tg brains that had received vehicle instead of IFNβ (Fig. 5a,b,d).

HIVgp120tg mice displayed significantly increased immunoreactivity for astrocytic GFAP compared to WT controls and intranasal IFNβ treatment did not significantly alter the immunofluorescence signal for GFAP except for a slight increase in the hippocampus of WT mice (Fig. 5a,b,e).

The number of Iba1-positive microglia also was significantly elevated in HIVgp120tg compared to WT control brains. IFNβ treatment did not change the cell count for microglia in the cortex of either WT or gp120tg mice (Fig. 5a,f), but led to a significant reduction of microglia in the hippocampus of gp120tg mice.

Altogether, we concluded that intranasal delivery of IFNβ rescued neurons from gp120-induced injury and reduced the associated microgliosis in the hippocampus, but not cortex, without reducing astrocytosis in either brain structure.

### Intranasal IFNβ treatment triggers expression of ISG in the brain

**Figure 6:**
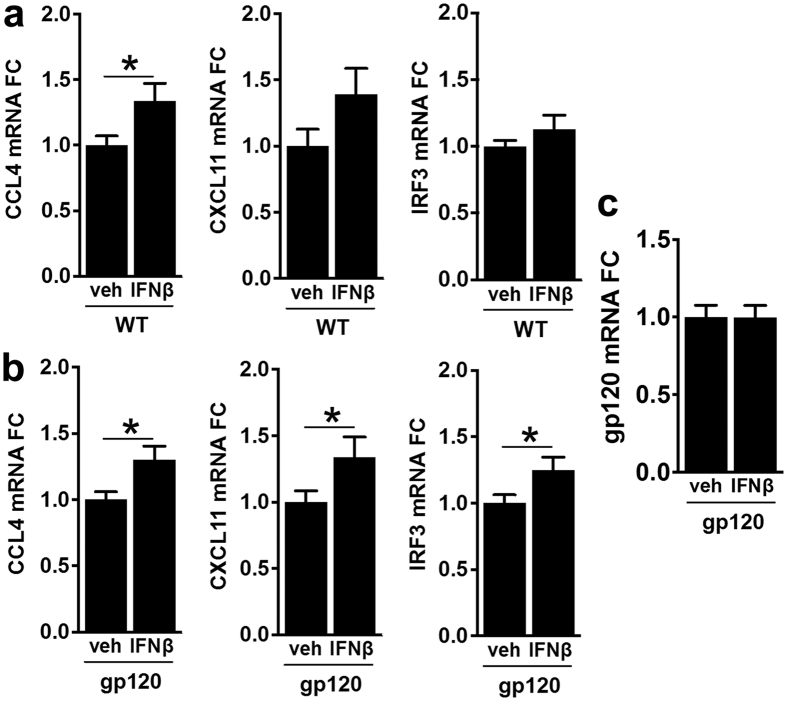
Intranasal IFNβ treatment triggers expression of ISG in the brain. RNA was purified from one brain hemisphere each of 4–5 month-old HIVgp120tg and WT littermate mice previously treated with intranasal IFNβ or vehicle and analyzed by qRT-PCR for fold-change (FC) in ISG expression. Significant changes in gene expression were observed between IFNβ and vehicle treatment groups in WT brains (**a**) for CCL4, and in gp120tg brains (**b**) for CCL4, CXCL11 and IRF3. Expression of transgenic HIVgp120 was not affected by IFNβ (**c**). Values are mean ± s.e.m.; n = 4–5 animals per group/genotype; *p < 0.05, student’s t-test.

Next, we investigated whether intranasally-delivered IFNβ caused significant changes in ISG expression in HIVgp120tg and WT brains. We analyzed changes in brain mRNA expression levels of CCL4, CXCL11 and interferon regulatory factor 3 (IRF3). CXCL11 was chosen as a marker of successful delivery because it has been reported to be prominently induced by IFNβ^48^. IRF3 is a key transcriptional regulator of the IFNα and β genes, as well as a direct activator of many IFN-stimulated genes involved in establishing an antiviral state^49^. CCL4 played a crucial role in protection by IFNβ against gp120 neurotoxicity *in vitro*. After the 4 week intranasal treatment with IFNβ, mRNA levels of CCL4, CXCL11 and IRF3 were all significantly elevated in HIVgp120tg brains whereas in WT the upregulation reached significance only for CCL4 (Fig. 6a,b). Although CXCL11 showed in response to IFNβ an increase of the mean mRNA level similar to CCL4, that increase was more variable and did not reach significance. However, expression of HIVgp120 remained unaffected by intranasal IFNβ application (Fig. 6c). Overall, upregulation of the ISG in HIVgp120tg mice was indicative of efficient delivery of IFNβ to the brain while we could also rule out that neuroprotection resulted from down-regulation of gp120 expression.

### Localization of CCL4 in astrocytes and neurons

**Figure 7:**
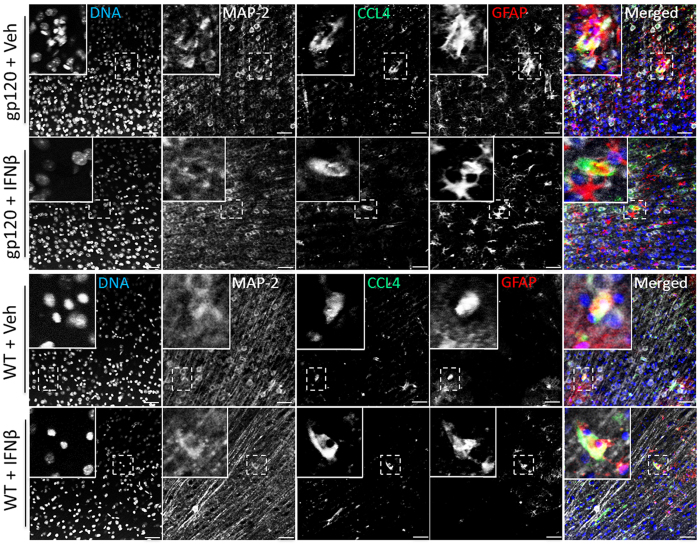
Localization of CCL4 *in vivo* in astrocytes and neurons. Sagittal brains sections of HIVgp120tg and WT littermate mice previously treated with intranasal IFNβ or vehicle (veh) were immunolabeled for CCL4, neuronal MAP-2 or astrocytic GFAP. Alexa Fluor 488, 555 and 647 conjugated secondary antibodies were employed to visualize primary Abs and nuclear DNA was labeled with Hoechst (H) 33342. The fluorescence-labeled brain sections were analyzed using confocal laser-scanning microscopy. Representative images of cortex layer III are shown; scale bar, 50 μm.

Since CCL4 appeared to play a crucial role in protection by IFNβ against gp120-induced neuronal injury *in vitro* and was up-regulated after intranasal IFNβ treatment with *in vivo*, we next attempted to visualize the β-chemokine in the mouse brain. Sagittal sections of brains from IFNβ-treated HIVgp120tg and WT mice were immunolabeled for CCL4 and cellular markers. Representative confocal microscopy images of layer III in the frontal cortex showed that with or without IFNβ treatment, CCL4 co-localized with GFAP-positive astrocytes and also MAP-2 positive neurons in HIVgp120tg and WT brains (Fig. 7).

### Interaction of IFNβ with neurons and astrocytes suffices to protect against neurotoxicity of HIVgp120-stimulated macrophages

**Figure 8:**
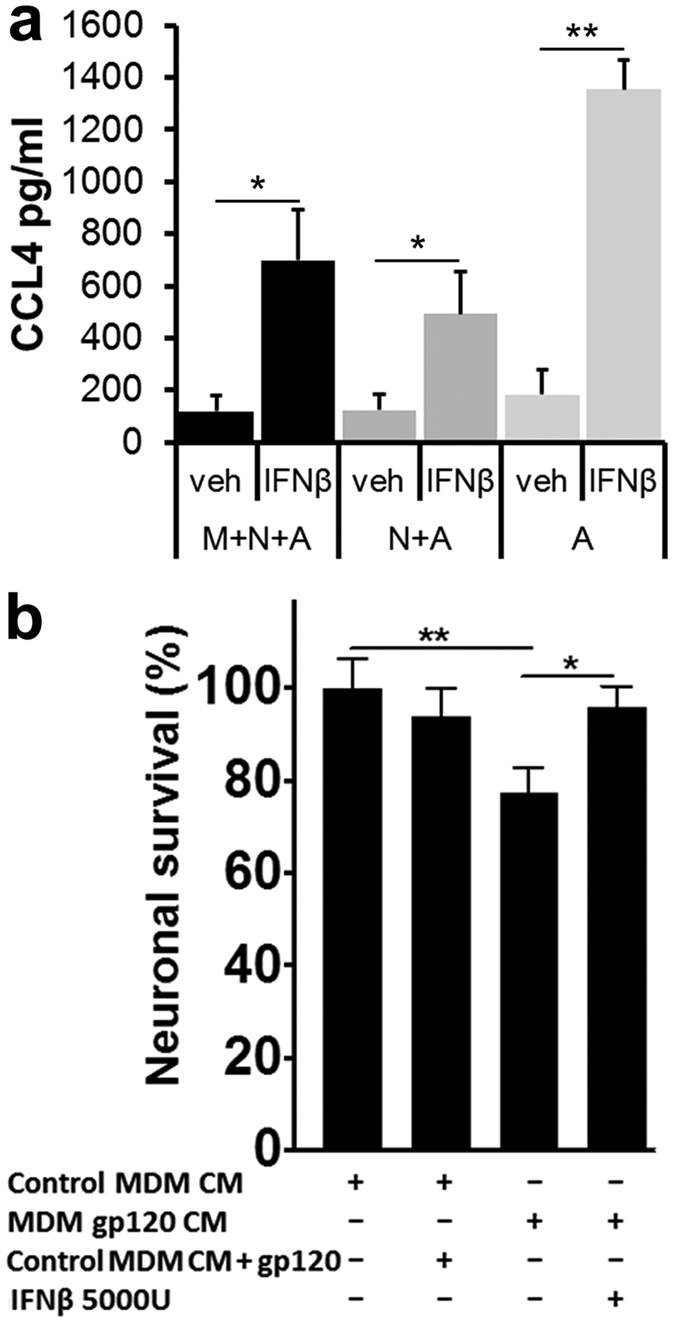
Interaction of IFNβ with neurons and astrocytes suffices to protect against neurotoxicity of HIVgp120-stimulated macrophages. (**a**) Cerebrocortical cultures from mice were prepared to either contain microglia, neurons and astrocytes (M + N + A) or were depleted of microglia (N + A) or neurons and microglia (A). Complete and depleted cell cultures were incubated with mIFNβ (5,000 U/ml) or BSA/PBS vehicle control for 0, 3, 6, 12 and 24 h and concentrations of CCL4 were measured in cell-free supernatants using a commercially available multiplex assay as described in Methods. Maximum concentrations were reached in samples of 12 to 24 h mIFNβ exposure and compared to vehicle-treated, baseline samples. Values are mean ± s.e.m.; n = 3 independent experiments; *p < 0.05, student’s t-test. (**b**) Microglia-depleted rat cerebrocortical cultures were exposed for 24 h to 50% cell-free conditioned media (CM) from human MDM in the presence or absence of human IFNβ (5,000 U/ml). MDM were previously stimulated for 24 h with HIV-1 gp120_BaL_ (MDM gp120 CM) or vehicle (MDM CM). Following the incubation the cells were fixed and permeabilized. Neurons were immunolabeled for neuronal MAP-2 and NeuN and nuclear DNA was stained with H33342. Neuronal survival was assessed using fluorescence microscopy and cell counting as described in Methods. Values are mean ± s.e.m.; n = 2 independent experiments, with 4–8 replicates and an average of 4,000 cells counted per condition; **p < 0.01, *p < 0.05 by ANOVA with Fisher’s PLSD post hoc test.

To investigate how neurons and astrocytes might contribute to the production of CCL4 in response to IFNβ, we returned to the *in vitro* system of cerebrocortical cells. We depleted the cell cultures of microglia in order to obtain cultures only containing neurons and astrocytes, or depleted both neurons and microglia to retain only astrocytes as described in the Method section^50^,^51^,^52^. Of note, the complete and depleted cell cultures in each of the experiments were derived from the same respective cell culture preparation and all samples, complete and depleted, were incubated in their respective conditioned culture medium with identical volumes. Therefore, microglia-depleted cultures contained the same quantity of neurons and astrocytes than their complete counterpart, and cell cultures depleted of neurons and microglia contained the same quantity of astrocytes than complete and microglia-deficient samples. Cerebrocortical cultures consisting of all three cell types, as well as neuronal-astrocytic and astrocyte cultures were then stimulated with IFNβ at 5,000 U/ml for 3, 6, 12 or 24 h and CCL4 protein was quantified in culture supernatants (Fig. 8a). Analysis of the maximal CCL4 concentrations secreted during 24 h indicated that depletion of microglia or neurons did not significantly affect the baseline expression of CCL4 compared to complete cultures. The up-regulation of CCL4 secretion in response to IFNβ was similar in complete and microglia-depleted cerebrocortical cultures but was higher and most pronounced in astrocyte cultures. This finding supported the notion that astrocytes were the major source of CCL4 in response to IFNβ exposure but also suggested that neurons contributed to regulating CCL4 released into the culture supernatant.

We and others have recently shown that cell-free conditioned media of human monocyte-derived macrophages stimulated with HIVgp120 caused neurotoxicity when transferred into microglia-depleted cerebrocortical neuroglial cell cultures of human, rat or mouse origin^50^,^53^,^54^,^55^,^56^,^57^. Since virtually all cell types in the CNS express type I IFN receptors and thus can interact with IFNβ, we assessed whether the interaction of IFNβ with astrocytes and neurons alone can be neuroprotective against gp120-stimulated macrophage toxins. Cerebrocortical cultures from rat were depleted of microglia as described in Methods and incubated in 50% cell-free conditioned media (CM) from gp120-exposed human monocyte derived macrophages (MDM) for 24 h, in the presence or absence of 5,000 U/ml recombinant human IFNβ, which has been shown to be fully active in the rat system^58^. Neuronal survival was significantly reduced in microglia-depleted cerebrocortical cells incubated with CM of MDM exposed to HIVgp120. However, the presence of IFNβ provided neuronal protection from toxins present in the CM of gp120-stimulated MDM (Fig. 8b). Due to the depletion of microglia, gp120 added to CM of un-stimulated MDM did not cause neurotoxicity, confirming that the neurotoxicity required the interaction of gp120 with MDM. Altogether, the data demonstrated that IFNβ interaction with neurons and astrocytes was sufficient for neuronal protection in the presence of MDM-derived toxins.

## Discussion

HIV-1 infection continues to cause HAND in the era of cART^3^,^59^. However, the viral infection does not immediately lead to overt disease as it triggers the host’s immune system, including the IFN response^21^. This early immune response temporarily controls the infection and thus appears to delay progression to HAND/HIVE and AIDS^21^. Here we show that HIVgp120tg mice, expressing the viral envelope protein in their CNS, mount a transient IFNβ response in their brains, and we provide evidence that IFNβ confers neuronal protection, at least, against toxicity of viral gp120.

Transient expression of IFNβ has also been observed in the CNS of SIV-infected macaques in association with extended viral control and delayed progression to disease in the brain^28^,^60^, suggesting that the HIVgp120tg mouse model recapitulates a relevant aspect of brain injury by lentiviral infection.

Although both type I IFNs signal through the same receptor complex consisting of IFNAR1 and 2, IFNβ and IFNα promote different biological responses in the CNS^61^. Expression of IFNβ is associated with an anti-inflammatory response in the CNS^33^,^34^,^62^, and as such is an FDA approved treatment for MS. In contrast, IFNα has been associated with neuroinflammatory and degenerative diseases, ranging from HIV-associated brain injury and HAND^25^,^26^ to Aicardi-Goutierres syndrome and Cree encephalitis^63^,^64^. Accordingly, transgenic expression of IFNα in the CNS of mice activates a progressive inflammatory encephalopathy and neurodegeneration^65^.

Studies in acute SIV infection in macaques showed that IFNβ is the main type I IFN produced by the brain^28^. The study linked an early IFNβ response to the translation of a truncated dominant negative isoform of C/EBP-β, which acts on long terminal repeats (LTR) of SIV to inhibit viral transcription^66^. In the classical model of type I IFNs signaling, production of IFNβ also leads to production of IFNα^67^. However, during SIV infection, brains induce a protective anti-viral response through the production of IFNβ, without activation of IFNα^68^. This regulation is mediated by CCL2, a β-chemokine which is produced predominantly by astrocytes upon viral infection. In SIV infection, CCL2 binds to the CCR2 receptor on macrophages to selectively suppress IFNα, without altering expression of IFNβ and anti-viral ISGs, such as MX1^69^. CCL2 is up-regulated in the brain of HIVgp120tg mice (Fig. 1f and ref. 15). Therefore, the absence of a concomitant increase in IFNα in the brains of HIVgp120tg mice at 1.5 months could be attributed to the upregulation of CCL2. Together with the reports on the SIV macaque model, our data provides evidence of differential type I IFN-signaling during expression of lentiviral envelope in the CNS, and suggests that HIVgp120tg brains can induce an IFNβ response without activation of neurotoxic IFNα. This preferential production of IFNβ rather than IFNα might reflect the host’s effort to suppress gp120-induced inflammation in order to protect neurons.

Although it is yet not known how HIVgp120tg mice mount the endogenous IFNβ response, the viral RNA sensor RIG-I is significantly upregulated in HIVgp120tg brains^15^. RIG-I is a host sensor for HIV RNA, including a section of the sequence that codes for gp120, and activates the type I IFN response^70^. RIG-I is expressed in microglia, astrocytes and neurons^71^,^72^, and therefore might be responsible for triggering endogenous IFNβ production at 1.5 months in the brains of HIVgp120tg mice.

However, why IFNβ transcription eventually returns to baseline levels in the brains of HIVgp120tg mice and SIV-infected macaques is unclear. Several possible explanations include, the down-regulation of cell surface IFNAR expression, induction of negative regulators, such as Suppressor of Cytokine Signaling (SOCS)-3 or ubiquitin carboxy-terminal hydrolase 18 (USP18), and the induction of miRNAs^73^. Studies in the SIV macaque model correlated SOCS3 expression patterns with an increase in CNS viral load and onset of disease^60^. Since SOCS3 expression is upregulated over time in HIVgp120tg brains^15^, it is possible that the viral gp120 is mediating suppression of endogenous IFNβ, which eventually results in failure to prevent brain injury.

The hypothesis that IFNβ can be neuroprotective against gp120-induced toxicity independently of suppressing viral infection is supported by the results of our *in vitro* and *in vivo* experiments. Intranasal administration of proteins and small molecules for therapeutic purposes, such as IGF-1 and estrogen, respectively, has been shown to be an effective method to bypass the blood brain barrier, delivering the drugs directly to the brain whilst reducing the potential risk of adverse effects in the periphery^74^,^75^,^76^. Drugs applied to the nasal cavity reach many areas in the CNS within minutes along olfactory and trigeminal nerves and via extracellular pathways which do not require ligand-receptor interactions or axonal transport^46^,^76^,^77^. Others showed in rats and monkeys that intranasal administration can deliver physiologically relevant amounts of intact IFNβ to the brain, including cerebral cortex, hippocampus and basal ganglia, areas strongly affected by HIV infection^46^,^47^. We found that neuronal injury in HIVgp120tg brains could be largely abrogated with a four-week intranasal IFNβ treatment (Fig. 5c,d). Although astrocytosis and increased numbers of microglia are hallmarks of HIV-1 and gp120 induced neuropathology, reversing or preventing these effects seems not to be a prerequisite for full neuronal protection. This is evidenced by our previous observations of increased neuronal survival independent of astrocytosis and microgliosis using EPO and IGF-1 in HIVgp120tg mice^45^ as well as overall unchanged astrocytosis in HIVgp120tg mice deficient in CCR5^15^. Studies in primary human fetal microglia showed that IRF3, a transcription factor required for the induction of IFNβ, facilitates a switch of microglial phenotype from pro- to anti-inflammatory by activating the PI3K/AKT pathway^78^. Interestingly, intranasal IFNβ treatment of gp120tg mice triggered an increase in IRF3 mRNA levels, thus it is possible that IFNβ treatment promoted a protective microglial phenotype.

Our *in vitro* and *in vivo* experiments also indicated a critical role of CCL4 in IFNβ protection. *In vitro*, IFNβ-stimulated cerebrocortical cells showed a significant induction of CCL4 at the mRNA and protein level (Fig. 3b,f). In addition, intranasal delivery of IFNβ significantly increased CCL4 mRNA in brains (Fig. 6a,b), and CCL4 protein was visualized in astrocytes and neurons of gp120tg as well as WT brains (Fig. 7). Moreover, experiments in cerebrocortical cell cultures using neutralizing antibodies against CCL4 abolished IFNβ-mediated neuroprotection, whereas antibodies against CCL3, CCL5 and CXCL10 (and IFNγ) had no effect (Fig. 4a). Finally, treatment with recombinant CCL4 alone protected cerebrocortical neurons against gp120-induced toxicity (Fig. 4c)^31^,^32^. Given the complexity of IFN responses, it seems unlikely that CCL4 and IRF3 are the only important contributors to IFNβ-mediated neuronal rescue from gp120-toxicity. Our studies in cerebrocortical cell cultures confirmed that IFNβ-protection is dependent on IFNAR1-signaling and results in induction of several CCR5 ligands and other ISGs, such as CXCL10. Earlier studies implicated the natural CCR5 ligands in slowing the progression of HIV infection and possibly HAND^29^,^32^. Our study characterized the effects of various IFNβ concentrations on the production of these chemokines in mixed-neuronal glial cerebrocortical cultures, both at RNA and protein levels in culture supernatants. Interestingly, mRNA levels did not predict protein levels. For example, CCL5 mRNA levels were highly upregulated by IFNβ treatment, however only week induction was observed at the protein level. The delay of CCL5 protein production post IFNβ treatment may explain why neutralizing antibodies against CCL5 did not abrogate the protective effects of IFNβ, and may indicate that IFNβ-mediated induction of CCL4 could play an important role in neuronal protection in acute HIV-infection. Overall, mRNA analysis showed concentration dependent induction of all four chemokines by IFNβ, but the amount of CCL3 and CCL4 protein detected in supernatants was similar between 500, 1000 and 5,000 U/ml of IFNβ, suggesting a potential feed-back mechanism that interferes at the level of protein translation or release. However, in contrast to 5,000 U/ml, 500 U/ml provided only partial protection of neurons against gp120 toxicity, suggesting other IFNβ-dependent factors may be involved.

Several lines of evidence indicate that microglia and macrophages play a crucial role in HIV infection and gp120-induced neurotoxicity, and we and others have shown that cell-free conditioned media of human MDM stimulated with HIVgp120 cause significant neurotoxicity when transferred into microglia-depleted cerebrocortical cell cultures of human, rat or mouse origin^31^,^50^,^53^,^54^,^55^,^56^,^57^. However, our *in vitro* experiments showed that the interaction of IFNβ with astrocytes and neurons alone was sufficient to protect neurons against gp120-induced toxicity of MDM (Fig. 8b). Moreover, our findings suggested that astrocytes might be major mediators of neuroprotective IFNβ effects by releasing protective CCL4. However, further studies are required to elucidate why IFNβ-induced chemokine release is substantially lower in the presence of neurons.

In summary, our study showed that IFNβ confers neuronal protection against toxicity of HIVgp120 via a pathway that involves CCL4. Moreover, intranasal treatment with IFNβ rescued neurons from injury in HIVgp120tg mice. Our findings indicate the biological relevance of a temporary endogenous IFNβ response in association with the expression of HIV-1 envelope gp120 in the brain and show the feasibility of treatment with exogenous IFNβ. Supplementary Fig. S4 provides a diagram summarizing our study. Moreover, since IFNβ is an FDA approved treatment of MS, application of exogenous IFNβ seems suitable to be tested in an infectious model, such as SIV-infected macaques, and if successful there, in HIV/HAND patients.

## Methods

### Reagents

Carrier-free, recombinant murine IFNβ (mIFNβ) produced in CHO cells was generously provided by PBL Assay Science (formerly PBL Interferon Source, Piscataway, NJ, cat# 12410). Recombinant human IFNβ (hIFNβ) originally from Avonex (Research Triangle Park, NC) was kindly provided by Dr. Sumit Chanda’s laboratory (Sanford Burnham Prebys Medical Discovery Institute, La Jolla, CA). Recombinant murine CCL4 was purchased from PeproTech (Rocky Hill, NJ, cat# 25032). Carrier-free, recombinant gp120 of the macrophage-tropic HIV-1 strain BaL was obtained from the National Institutes of Health AIDS Research and Reference Reagent Program, Division of AIDS, NIAID. All cytokines and recombinant gp120 were reconstituted in 0.1% BSA/PBS (at 100 x final concentration). Bacterial lipopolysaccharide (LPS) was purchased from Sigma (St. Louis, MO, cat# L6529) and was sonicated before use. Neutralizing antibodies against the following mouse proteins were purchased from R&D Systems (Minneapolis, MN) and reconstituted in sterile PBS: CCL3 (cat# AF450NA), CCL4 (cat# AF451NA), CCL5 (cat# AF478NA), CXCL10 (cat# AF466NA), IFNγ (cat# AF585NA). All antibodies were tested for neutralizing activity, function and endotoxin level by the manufacturer’s Quality Assurance program. All reagents were stored in aliquots according to the manufacturer’s recommendations at 4, −20 or −80 °C until use.

### Animals

HIVgp120tg mice were kindly provided by Dr. Lennart Mucke (Gladstone Institute of Neurological Disease, University of California, San Francisco, CA)^13^. Two founder lines of HIVgp120tg mice (L1 and L2) were recently characterized in terms of CNS gene expression and the role of CCR5 in brain injury associated with the expression of the viral envelope protein^15^. Mice deficient in functional IFNAR1 (B6.129S2-*Ifnar1*^*tm1Agt*^)^42^ were kindly provided by Dr. Carl Ware (Sanford Burnham Prebys Medical Discovery Institute, La Jolla, CA). Genotyping of mice used genomic DNA isolated from tail clippings and employed protocols published in the literature^13^ and by The Jackson Laboratory. For some experiments, timed pregnant Sprague-Dawley rats were obtained from Harlan Labs Inc. (San Diego, CA) in order to prepare mixed neuronal/glial cerebrocortical cell cultures. All experimental procedures and protocols involving animals were in accordance with NIH guidelines and approved by the Institutional Animal Care and Use Committee of the Sanford Burnham Prebys Medical Discovery Institute.

### Bioinformatics

The list of 1,195 genes differentially expressed in the brain of HIVgp120tg mouse line 2 (L2) and the respective microarray data have been published recently^15^. The gene list and relative expression values were uploaded into the commercially available bioinformatic tools of Ingenuity Pathway Analysis (IPA, Ingenuity^®^ Systems, www.ingenuity.com; build version: 389077 M; content version: 27821452; release date: 2016–06–14) and interrogated for upstream regulators using the Core Analysis function as previously described^15^,^79^. Details of analysis settings are described in Supplementary Methods.

### Cerebrocortical cell cultures

Mixed cerebrocortical cell cultures containing neurons, astrocytes, and microglia were prepared from embryos of Sprague-Dawley rats at day E15-17 as previously described by our group^31^,^32^,^50^,^51^,^52^. Murine mixed cerebrocortical cultures were prepared from E14.5 embryos of non-tg, wild-type control or IFNAR1KO mice, as described previously with minor modifications^15^,^32^. For neurotoxicity experiments, cells were seeded at 6.5 x 10^6^ per 96-well plate and at 1.1 x 10^7^ per 24-well plate for mRNA and protein expression experiments. Rat and murine cerebrocortical cells were typically used at day 14–17 *in vitro*. For experiments in which we studied the contribution of microglia, astrocytes or neurons to CCL4 production, neurons were depleted by treatment with 300 μM *N*-methyl-*D*-aspartate (NMDA, Abcam, Cambridge, MA, cat#ab120052) 3 days prior to the experiment^51^,^52^. For experiments that required microglial depletion, cerebrocortical cells were pre-treated with 7.5 mM L-leucine-methyl ester (LME) for 24 h (rat)^50^,^51^,^52^ or 4 h (mouse) prior to experimental treatments^15^,^50^. Detailed protocols are described in Supplementary Methods.

### Preparation of MDM and cell-free neurotoxic conditioned media

Human primary monocyte-derived macrophages (MDM) were prepared using Ficoll gradient centrifugation as previously described with minor modifications^50^. A detailed protocol is described in Supplementary Methods. To produce neurotoxic conditioned cell-culture supernatants, 7–8 day old primary MDM were transferred into media for rat cerebrocortical cells and incubated with recombinant viral envelope gp120 of HIV strains BaL (200 pM) or BSA/PBS vehicle alone as control (at 0.001% final concentration). Following 24 h stimulation, cell-free supernatants were at 50% concentration transferred onto the cerebrocortical cells previously depleted of microglia. Neuronal survival was assessed after 24 h of incubation.

### Neurotoxicity assay

Cerebrocortical cell cultures from rat or mouse were exposed for 24–72 hrs to recombinant gp120 of HIV-1 strain BaL (200 pM) in the presence or absence of recombinant mIFNβ or hIFNβ (500 to 5,000 U/ml) or mCCL4 (2 or 20 nM) or neutralizing antibodies (1 μg/ml). Controls cells were exposed to 0.001% BSA/PBS vehicle. The Neutralization Dose (ND50) for antibodies against murine CCL3, CCL4, CCL5, CXCL10 and IFNγ was provided by the manufacturer (R&D Systems) and each antibody was added to the cell culture medium at ≥10 times the concentration required to neutralize the protein concentration in culture supernatants determined with Multiplex assays (Fig. 3). Neuronal survival and loss in cerebrocortical cell cultures was assessed after experimental treatments using immunolabeling of neuronal MAP-2 and NeuN and nuclear DNA staining as described earlier^15^,^32^,^50^,^51^,^52^. Neuronal survival was assessed using fluorescence microscopy and calculated from the number of MAP-2 and NeuN double-positive cells in the total cell number and vehicle-treated control samples were defined as 100% survival. A detailed protocol is described in Supplementary Methods.

### Multiplex assays of cytokines and chemokines

For protein analysis, cell culture supernatants were collected on ice from mouse cerebrocortical cell cultures at the indicated time points (3 to 24 h) post-treatment with mIFNβ (500–5,000 U/mL) or BSA/PBS as vehicle control (indicated as 0 h exposure to IFNβ). Samples were centrifuged to remove cell debris and analyzed for 6 different proteins (CCL3, CCL4, CCL5, CXCL10, IFNβ and IFNγ) using a commercially available Milliplex Mouse Cytokine/Chemokine Magnetic Bead Panel from Millipore (Billerica, MA, cat # MCYTOMAG-70K) following the supplier’s instructions. Bead-bound protein concentrations were measured with MAGPIX system from Millipore and analyzed by Milliplex Analysis (Millipore, Billerica, MA).

### Intranasal treatment of HIVgp120tg mice with IFNβ

Generally, intranasal treatment of mice was performed as described previously^45^. Briefly, male HIVgp120tg and WT littermate control mice were randomly divided into two treatment groups: IFNβ (50,000 U/25 g body weight) and a vehicle-treated (375 μg mouse serum albumin/ml in 0.375% gelatin in sterile PBS) control group. The animals were anesthetized with 2.5–3% of isoflurane in oxygen (flow rate at 0.8 liters/min) and maintained under anesthesia (1.5–2% of isoflurane and oxygen flow to 0.8 liters/min) throughout the intranasal treatment. Body temperature was maintained at 37 °C using a heating pad. For intranasal IFNβ delivery, the animal was placed in a supine position with its neck slightly elevated by rolled-up gauze, and IFNβ or vehicle alone was administered dropwise in 3 μl aliquots into the nares with an Eppendorf pipette. Aliquots were delivered to alternate nostrils every 2 min, over the course of 12 min. Treatments were given once a week for 4 weeks for a total of four doses. During the experimental treatment, all mice were monitored weekly for body weight and general appearance (color and structure of fur, posture, alertness). Within 4 h of the last treatment animals were terminally anesthetized, and following cardiac perfusion with 0.9% saline solution, the brains were collected. Brains were hemisected, with one half snap frozen for RNA and the other fixed in 4% PFA in PBS for immunohistological analysis.

### Immunohistology and quantitative fluorescence microscopy

Neuropathological analysis using 30 μm thick sagittal brain sections, deconvolution and quantitative fluorescence microscopy was performed as previously published with minor modifications^15^,^79^. Slidebook software (version 5 and 6, Intelligent Imaging Innovations, Inc., Denver, CO) was used for image acquisition and analysis. A detailed protocol is described in Supplementary Methods.

For visualization of CCL4, mouse brain sections were immunolabeled with primary antibodies for CCL4 (ProSci, Poway, CA, cat# 7227, 1:50) in combination with Ab against MAP-2 or GFAP for 24 h. Alexa Fluor 488, 555 and 647 conjugated secondary antibodies were employed to visualize primary Abs and nuclear DNA was stained with Hoechst (H) 33342. Images were acquired with a laser-scanning microscope (Zeiss LSM710) and processed and pseudo-colored using ImageJ and Adobe Illustrator software.

### RNA isolation and quantitative RT-PCR

Isolation of total RNA from mouse brain and cerebrocortical cell cultures as well as qRT-PCR were performed and analyzed using the ∆∆Ct approach as previously described^15^,^79^. Briefly, following treatment, cerebrocortical cells were transferred onto ice, quickly washed once with cold 1 x PBS and total RNA was purified using an RNeasy Mini Kit isolation kit (Qiagen, Valencia, CA, cat# 74104), according to the manufacturer’s instructions. Total mouse brain RNA was isolated using the Qiagen RNeasy Lipid Tissue Midi Kit (Qiagen, cat# 75144). A list of the qRT-PCR primers is provided in Table 1.

### Statistical analysis

Experimental results are shown as mean values ± s.e.m. Comparisons of more than two experimental groups employed analysis of variance (ANOVA) followed by Fisher’s PLSD post hoc test. Student’s t-test was used for comparison of two experimental conditions. All statistical analysis was performed using StatView software (version 5.0.1, SAS Institute, Cary, NC) and the significance level was set at p = 0.05.

## Additional Information

**How to cite this article:** Thaney, V. E. *et al*. IFNb Protects Neurons from Damage in a Murine Model of HIV-1 Associated Brain Injury. *Sci. Rep.* **7**, 46514; doi: 10.1038/srep46514 (2017).

**Publisher's note:** Springer Nature remains neutral with regard to jurisdictional claims in published maps and institutional affiliations.

## Supplementary Material

Supplementary Information

## Figures and Tables

**Table 1:** Primers for qRT-PCR.

Gene	GenBank	References	Primer Sequence (5′-3′)
mCcl2/ Mcp1	NM_011333.3	15	Fwd: CCCAATGAGTAGGCTGGAGARev: TCTGGACCCATTCCTTCTTG
mCcl3/Mip1α	NM_011337.2	15	Fwd: GCGCCATATGGAGCTGACARev: GATGAATTGGCGTGGAATCTTC
mCcl4/Mip1β	NM_013652.2	15	Fwd: AGGGTTCTCAGCACCAATGGRev: AGCTGCCGGGAGGTGTAAG
mCcl5/Rantes	NM_013653.3	15	Fwd: ACACCACTCCCTGCTGCTTTRev: TGCTGCTGGTGTAGAAATACTCCTT
mCxcl10	NM_021274.2	80	Fwd: GCCGTCATTTTCTGCCTCATRev: GGCCCGTCATCGATATGG
mCxcl11	NM_019594.1	81	Fwd: GGCTTCCTTATGTTCAAACAGGGRev: GCCGTTACTCGGGTAAATTACA
mMx1	NM_010846.1		Fwd: AGAGCAAGTCTTCTTCAAGGATCACRev: GTGGCCTTCCCATCTTCCA
mIrf3	NM_016849.4		Fwd: CAGATCTGATTGCCTTCATGGARev: ACATTTCCCCCATGCAGAAC
Gapdh	NM_008084.2	15	Fwd: AGGTCGGTGTGAACGGATTTGRev: TGTAGACCATGTAGTTGAGGTCA
mIfna	M_28587.1	82	Fwd: TGCAATGACCTCCATCAGCARev: TTCCTGGGTCAGAGGAGGTTC
mIfnb1	NM_010510.1		Fwd: GAAAGGACGAACATTCGGAAATRev: CGTCATCTCCATAGGGATCTTGA
mIFNγ	NM_008337.3	83	Fwd: TAGCTCTGAGACAATGAACGCTACRev: GTGATTCAATGACGCTTATGTTGT
HIV-1 gp120	M19921	15	Fwd: TGAGCCAATTCCCATACATTATTGRev: CCTGTTCCATTGAACGTCTTATTATTAC
