# Supplementary material for: IFNβ Protects Neurons from Damage in a Murine Model of HIV-1 Associated Brain Injury
Source: Sci Rep. 2017 Apr 20;7:46514. doi: 10.1038/srep46514 (PMC5397848; doi:10.1038/srep46514)
Supplement: Supplementary Information [file srep46514-s1.pdf]

Supplementary Information

IFNβ Protects Neurons from Damage in a Murine Model of HIV-1 Associated Brain Injury

Victoria E. Thaney[1,2], Alan O'Neill[1], Melanie M. Hoefer[1], Ricky Maung[1], Ana B. Sanchez[1], Marcus Kaul[1,3]

[1]Infectious and Inflammatory Disease Center, [2]Graduate School of Biomedical Sciences, Sanford-Burnham Prebys Medical Discovery Institute, 10901 North Torrey Pines Road, La Jolla, CA 92037, USA

[3]Department of Psychiatry, University of California, San Diego, 9500 Gilman Drive, San Diego, CA 92093, USA

Supplementary Figure S1

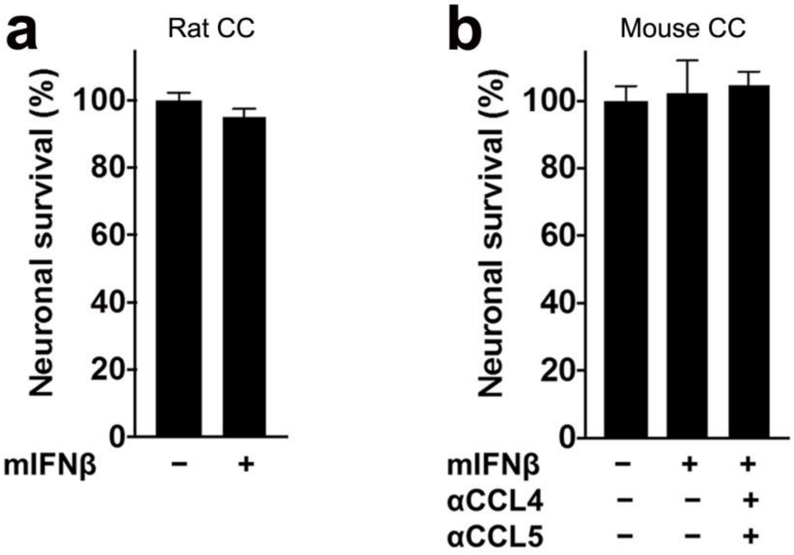

Legend to Supplementary Figure S1. Neuronal survival in cerebrocortical cell cultures (CC) from rat and mouse is not compromised by IFNβ. Neuronal-glial cerebrocortical cultures from rat (**a**) and IFNAR1 WT mice (**b**) were treated for 24 h or 3 days, respectively, with mouse IFNβ (5,000 U/ml). Mouse cerebrocortical were in addition exposed to IFNβ in the presence of a combination of neutralizing antibodies against CCL4 and CCL5 as described in Methods. Controls received vehicle. Following the incubation the cerebrocortical cultures were fixed and permeabilized, and neurons were immunolabeled for neuronal MAP-2 and NeuN whereas nuclear DNA was stained with H33342. Neuronal survival was analyzed using fluorescence microscopy and cell counting as described in Methods. Values are mean ± s.e.m.; n = 3 independent experiments with 4 - 7 replicates per condition. Student's t-test, $p = 0.14$ (**a**) and ANOVA, $p = 0.78$ (**b**) indicated no significant differences between the experimental conditions.

Supplementary Figure S2

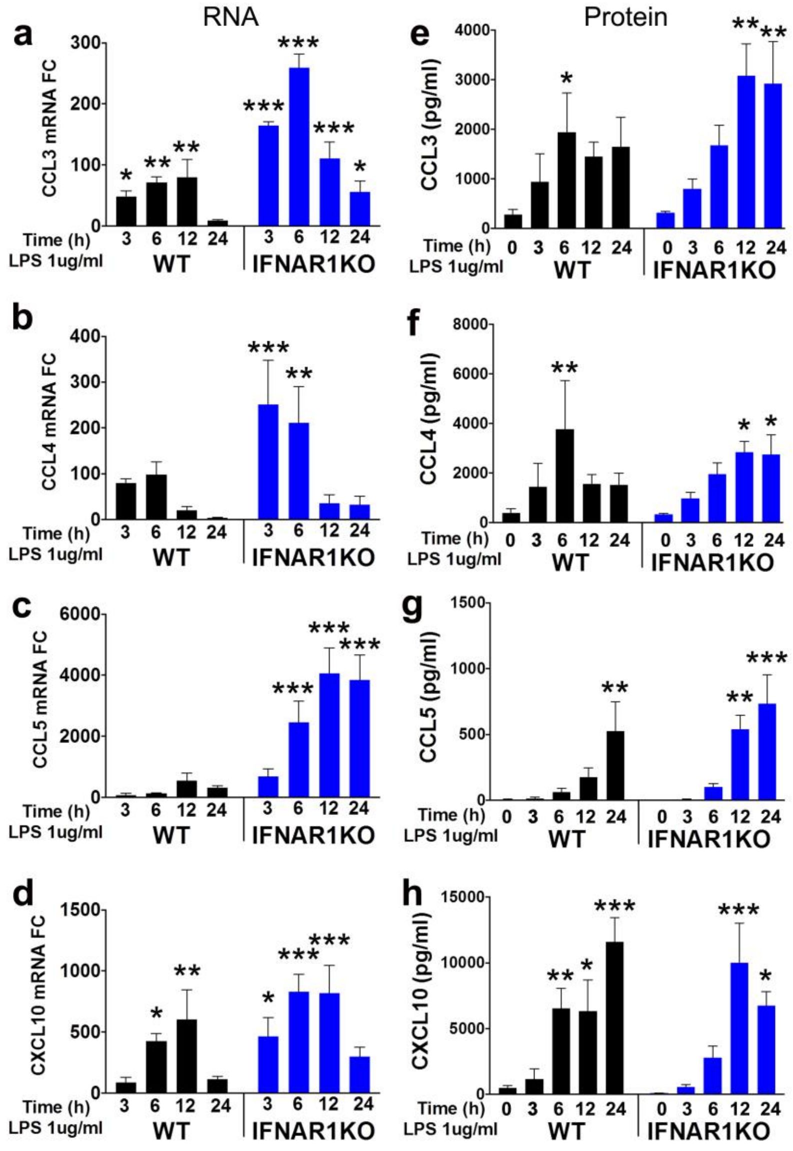

Legend to Supplementary Figure S2. IFNAR1 independent induction of ISGs by LPS in mouse mixed-cerebrocortical cultures. Neuronal-glial cerebrocortical cultures from WT and IFNAR1KO mice were treated with LPS (1 µg/ml) or vehicle control for 0, 3, 6, 12 and 24 h. Total RNA was extracted from cell lysates, analyzed by qRT-PCR and normalized to GAPDH expression levels. (**a - d**) RNA expression is shown as fold change (FC) in relation to vehicle treated controls which were defined as baseline activity. (**e - h**) Time course for protein expression measured in cell-free supernatants for CCL3, CCL4, CCL5 and CXCL10 using a commercially available multiplex assay as described in Methods. Baseline protein expression in vehicle treated cell cultures is represented as 0 h exposure time point. Values are mean ± s.e.m.; n = 3 independent experiments per ISG; *** $p < 0.001$, ** $p < 0.01$, * $p < 0.05$ by ANOVA with Fisher's PLSD post hoc test. For clarity, the significance is only indicated for differences between treatments and baseline within each experimental group.

Supplementary Figure S3

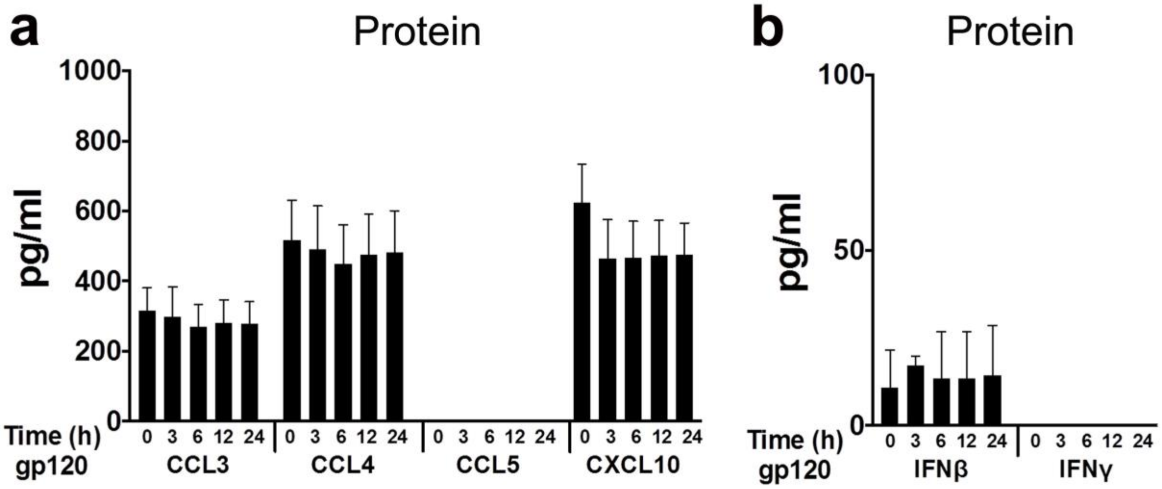

Legend to Supplementary Figure S3. Incubation of cerebrocortical cultures with HIVgp120 at concentrations that cause neurotoxicity (200 pM) fails to induce IFNs or ISG. Neuronal-glial cerebrocortical cultures from WT mice were treated with 200 pM gp120 or vehicle control for 0, 3, 6, 12 and 24 h. Cell-free supernatants were collected and protein concentrations were measured for (**a**) CCL3, CCL4, CCL5, CXCL10, (**b**) IFNβ and IFNγ protein using a multiplex assay as described in Methods. Baseline protein expression in vehicle-treated cell cultures is represented as the 0 h time point. Values are mean ± s.e.m.; n = 3 independent experiments per ISG, n = 2 for IFNs, each with 3 replicates per condition.

Supplementary Figure S4

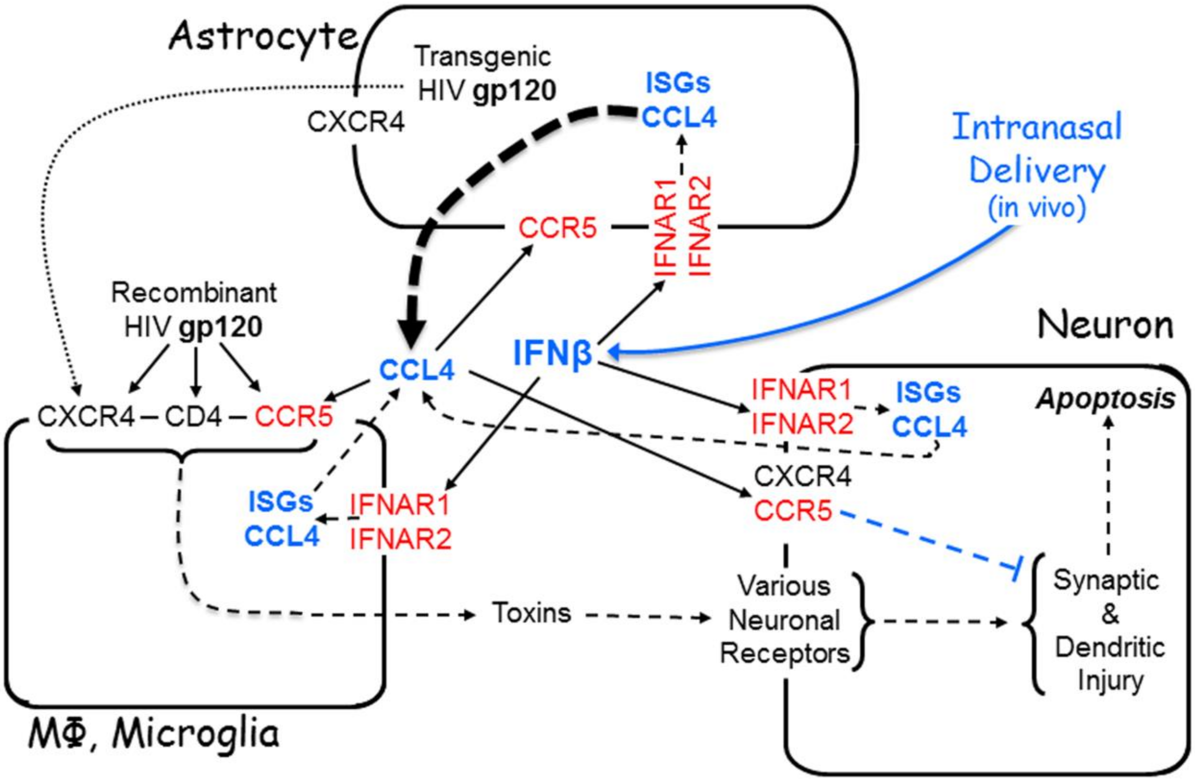

Legend to Supplementary Figure S4. Model for neuroprotection by IFNβ in HIV-induced neuronal injury. Stimulation with HIV-1 gp120 triggers in macrophages (MΦ) and microglia the release of neurotoxins. The viral protein and other toxins may all act in concert to cause neuronal injury and apoptosis. HIVgp120-transgenic (tg) mice transiently express endogenous IFNβ and numerous IFN-stimulated genes (ISGs) in their brains. Exogenous IFNβ applied to cerebrocortical cell cultures or intra-nasally delivered to HIVgp120tg mice protects neurons against the toxicity of HIVgp120 via a pathway that requires the β-chemokine and CCR5 ligand CCL4. IFNβ and CCL4 may directly affect neurons, astrocytes and MΦ/microglia via IFNAR1/2 (IFNα/β receptor) and CCR5, respectively, to provide neuroprotection, but the interaction of IFNβ with neurons and astrocytes suffices to abrogate neuronal injury and death caused by gp120-induced macrophage neurotoxins. Astrocytes appear to be the major source of CCL4 released in response IFNβ stimulation. Solid arrows indicate direct interactions whereas dashed arrows represent effects with additional intermediary steps not included in this diagram.

Supplementary Methods

Bioinformatics

The list of 1,195 genes differentially expressed in the brain of HIVgp120tg mouse line 2 (L2) and the respective microarray data have been published recently[1]. Of note, 734 of the 1,195 genes are differentially regulated only in the presence of both, gp120 and CCR5 whereas 461 genes are differentially regulated in HIVgp120tg brains independently of CCR5 expression. The combined list of 1,195 genes and the relative expression values were uploaded into the commercially available bioinformatic tools of Ingenuity Pathway Analysis (IPA, Ingenuity® Systems, www.ingenuity.com; build version: 389077M; content version: 27821452; release date: 2016-06-14). Using the Core Analysis function of IPA the gene list was evaluated for upstream regulators. The analysis settings were as follows: Cutoff before duplicate resolution: TRUE; Resolve duplicate on: fold change; Color using: fold change; Cutoff for fold change: --; Reference set: MouseWG-6 v2.0; Relationship to include: Direct and Indirect; Include Endogenous Chemicals: No; Optional Analyses: My Pathways, My List; Filter Summary: Consider only molecules and/or relationships where (confidence = Experimentally Observed) AND (tissues/cell lines = PBMCs OR Other Macrophages OR Monocyte-derived macrophage OR Natural T-regulatory cells OR Mast cells OR BDCA-3+ dendritic cells OR SF-539 OR CD34+ cells OR Bone marrow-derived dendritic cells OR Immature monocyte-derived dendritic cells OR Memory B cells OR Neutrophils OR Sciatic Nerve OR Lymphocytes not otherwise specified OR Gray Matter OR Subventricular Zone OR Corpus Callosum OR Spinal Cord OR Caudate Nucleus OR Pyramidal neurons OR Eosinophils OR Peripheral blood leukocytes not otherwise specified OR Other B lymphocytes OR Purkinje cells OR Other Monocytes OR Th1 cells OR Mononuclear leukocytes not otherwise specified OR Intraepithelial T lymphocytes OR

Tissues and Primary Cells not otherwise specified OR Cerebral Cortex OR Vd2 Gamma-delta T cells OR CNS Cell Lines not otherwise specified OR Amygdala OR Activated helper T cells OR Th17 cells OR Choroid Plexus OR Other Immune cells OR Langerhans cells OR Other Macrophage Cancer Cell Lines OR Immune cells not otherwise specified OR Mature monocyte-derived dendritic cells OR Effector T cells OR Pre-B lymphocytes OR Thalamus OR Murine NKT cells OR Activated Vd1 Gamma-delta T cells OR Other CNS Cell Lines OR RAW 264.7 OR Other T lymphocytes OR Other Memory T lymphocytes OR SNB-75 OR Other Peripheral blood leukocytes OR Peritoneal macrophages OR Central memory helper T cells OR Putamen OR SF-268 OR CD56bright NK cells OR Plasmacytoid dendritic cells OR Pituitary Gland OR Effector memory cytotoxic T cells OR B lymphocytes not otherwise specified OR Macrophage Cancer Cell Lines not otherwise specified OR SF-295 OR White Matter OR Other Lymphocytes OR Naive B cells OR Striatum OR Memory T lymphocytes not otherwise specified OR Vd1 Gamma-delta T cells OR Olfactory Bulb OR Other Mononuclear leukocytes OR Neurons not otherwise specified OR Cerebellum OR Microglia OR Other Dendritic cells OR Parietal Lobe OR Monocytes not otherwise specified OR Macrophages not otherwise specified OR Hippocampus OR Activated Vd2 Gamma-delta T cells OR Naive helper T cells OR NK cells not otherwise specified OR Cells not otherwise specified OR CD4+ T-lymphocytes OR BDCA-1+ dendritic cells OR Dorsal Root Ganglion OR Plasma cells OR Cytotoxic T cells OR Thymocytes OR Pro-B lymphocytes OR Nervous System not otherwise specified OR Trigeminal Ganglion OR Effector memory RA+ cytotoxic T cells OR Central memory cytotoxic T cells OR Other Granulocytes OR Astrocytes OR Granule cells OR Activated CD56bright NK cells OR Bone marrow-derived macrophages OR Myeloid dendritic cells OR Granule Cell Layer OR Activated CD56dim NK cells OR Other Monocyte-derived dendritic cells OR Peripheral blood monocytes

OR U251 OR Other Nervous System OR Peripheral blood lymphocytes OR Brainstem OR Ventricular Zone OR Monocyte-derived dendritic cells not otherwise specified OR Cerebral Ventricles OR Dendritic cells not otherwise specified OR Substantia Nigra OR Hypothalamus OR Other Neurons OR T lymphocytes not otherwise specified OR Granulocytes not otherwise specified OR Effector memory helper T cells OR Medulla Oblongata OR SNB-19 OR Brain OR Cortical neurons OR Other NK cells OR Nucleus Accumbens OR CD56dim NK cells OR Th2 cells.

Cerebrocortical cell cultures

Mixed cerebrocortical cell cultures containing neurons, astrocytes, and microglia were prepared from embryos of Sprague-Dawley rats at day 15-17 of gestation and cultured in poly-D-lysine-coated (PDL; Sigma, cat# P7280) flat-bottom plates for immunofluorescent imaging (0.087 x 10^6 cells per well) (BD Falcon, BD Biosciences, San Jose, CA, cat # 353219)[2-6]. D10C culture medium contained 77 % Dulbecco's modified Eagle's medium (DMEM) with high glucose (Thermo Fisher Scientific, Pittsburgh, PA, cat # 11960044), 9.6 % heat-inactivated horse serum (Thermo Fisher Scientific, cat# 26050088), 9.6 % F12 medium (Thermo Fisher Scientific, cat# 10565018), 2.4 % 1 M Hepes (Thermo Fisher Scientific, cat# 15630080), 1 % 200 mM L-glutamine (Thermo Fisher Scientific, cat# 25030081), and 0.2 % 100 U/ml penicillin with 100 μg/ml streptomycin (Thermo Fisher Scientific, cat# 15140122). The cells were typically used at day 14-17 *in vitro*, once the neurons were considered to be fully differentiated. For experiments that required microglial depletion, cells were pre-treated with 7.5 mM L-leucine-methyl ester (LME) for 24 h[4-6].

Murine mixed cerebrocortical cultures were prepared from E14.5 embryos of non-tg, wild-type control or IFNAR1KO mice, as described previously with minor modifications[1,3]. For neurotoxicity experiments, cells were seeded at 6.5 x 10^6 per 96-well plate and at 1.1 x 10^7 per 24-well plate for mRNA and protein expression experiments. Culture medium contained 87 % DMEM with high glucose, 10 % heat-inactivated horse serum, 1 % 1 M Hepes, 1 % 200 mM L-glutamine, and 1 % 100 U/ml penicillin with 100 μg/ml streptomycin. Murine cerebrocortical cells were typically used at day 14 - 17 *in vitro*, and for experiments in which we studied the contribution of microglia, astrocytes or neurons to CCL4 production, neurons were depleted by treatment with 300 μM *N*-methyl-*D*-aspartate (NMDA, Abcam, Cambridge, MA, cat#ab120052) 3 days prior to the experiment[5,6]. For some experiments murine microglia were depleted with 7.5 mM LME for 4 h prior to experimental treatments[1,4].

Isolation, preparation and cell-mediated neurotoxicity in MDMs

Human primary monocyte-derived macrophages (MDM) were prepared using Ficoll gradient as previously described with minor modifications[4]. In brief, buffy coat cells from anonymous healthy donors were provided by the San Diego Blood Bank (San Diego, CA, USA) and were further purified using density gradients of Ficoll-paque (GE Health Life Sciences, Pittsburgh, PA, cat#17089109) and cultured in RPMI 1640 medium (Hyclone, GE Health Life Sciences, cat#SH30096.01) containing 2 nM glutamine, 100 U/ml penicillin with 100 μg/ml streptomycin, 10 % human AB serum (RPMI-ABS) (Corning TM, Fisher Scientific, cat# 35060CI), 10 % FBS (Hyclone, GE Health Life Sciences, cat# SH30070.03) and transferred into 75 cm^2 cell culture flasks to incubated at 37 ˚C, 6 % CO2. Non-adherent cells were removed by rigorous washing with warm RPMI 1640 medium. Adherent monocytes were cultured for 5 days to allow for

differentiation into MDM. Next, cells were detached by treatment with PBS containing EDTA (final concentration 0.2 %) for 5 min at 37 ˚C and then scraped with a rubber policeman, washed three times with RPMI-ABS medium and finally reseeded at 10^6 cells/ 1 ml RPMI-ABS per well in 12-well plates.

Neurotoxicity assay

Neuronal survival and loss in cerebrocortical cell cultures from rat and mouse was assessed after experimental treatments as described earlier[1,3-6]. In brief, cerebrocortical cell cultures were exposed for 24 - 72 hrs to recombinant gp120 of HIV-1 strain BaL (200 pM) in the presence or absence of mouse IFNβ (500 to 5,000 U/ml) or human IFNβ (5,000 U/ml) or mCCL4 (2 or 20 nM) or neutralizing antibodies. Controls cells were exposed to 0.001 % BSA/PBS vehicle. The Neutralization Dose (ND50) for antibodies against murine CCL3, CCL4, CCL5, CXCL10 and IFNγ was provided by the manufacturer (R&D Systems) and each antibody (Ab) was added to the cell culture medium at ≥ 10 times the concentration required to neutralize the measured protein concentration in culture supernatants determined with Multiplex assays (Fig. 3) and at even protein concentration for all Abs (1. After the incubation, cells were fixed with 4 % PFA for 25 min and permeabilized with 0.2 % Triton X-100 for 5 min. Neurons were immunolabeled for specific markers with mouse anti-MAP-2 (Sigma, cat# M4403, 1:500) and rabbit anti-NeuN Ab (Millipore, Billerica, Massachusetts 01821, USA, cat# ABN78, 1:500). For control purposes, primary Abs were replaced with an irrelevant IgG of the same species and subclass. Secondary Abs included goat anti-mouse Rhodamine Red (Jackson ImmunoResearch, West Grove, PA, cat# 115295146, 1:200) and goat anti-rabbit Alexa Fluor 488 (Thermo Fisher Scientific, cat# A11034, 1:400). Nuclear DNA was stained with Hoechst (H) 33342 (Sigma, cat# B2261) at a final

concentration of 12 μM. Microscopy and cell counting was carried out using a Zeiss 200M fluorescence microscope (Zeiss) with filters for DAPI, FITC, and CY3. Generally, cells were counted in 2 - 3 fields each per 4 - 11 replicates per experimental condition of two or three independent experiments. Neuronal survival was calculated from the number of MAP-2 and NeuN double-positive cells in the total cell number and vehicle-treated control samples were defined as 100% survival.

Immunohistology and quantitative fluorescence microscopy

Histopathological analysis using 30 µm thick sagittal brain sections was performed as previously published with minor modifications[1,7]. Briefly, brain sections were prepared with a vibratome (Leica VT 1000S, Leica Biosystems, Buffalo Grove, IL), permeabilized and immunostained with primary Abs against mouse synaptophysin (SYP, Dako, Carpinteria, CA, cat# M7315, 1:50) and mouse MAP-2 (Sigma, cat # M4403, 1:125) as neuronal markers, or GFAP (Dako, Carpinteria, CA, cat# Z0334, 1:500) and Iba1 (Wako, Richmond, VA, cat# 01919741, 1:125) as astrocytic and microglial markers, respectively. For control purposes, primary Abs were replaced with mouse IgG1 (MOPC21, Sigma, cat# M9269, 1:500), or primary Abs were omitted. Secondary Abs were goat anti-mouse-Rhodamine Red (Jackson ImmunoResearch, cat # 115295146, 1:200) and goat anti-rabbit Alexa Fluor 488 (Thermo Fisher Scientific, cat# A11034, 1:400). Nuclear DNA was stained with Hoechst (H) 33342 (Sigma, cat# B2261) at a final concentration of 12 μM. Immunolabelled brain sections were mounted with fluorescence-protecting mounting medium (VectaShield, Vector laboratories, Burlingame, CA, cat# H1000) onto glass slides and covered with coverslips. Image acquisition and analysis was performed using a Zeiss 200M fluorescence deconvolution microscope equipped with a computer-aided Z-drive and stage and

with filters for DAPI, FITC, CY3 and CY5 (Carl Zeiss Microscopy GmbH, Jena, Germany). To assess neuronal injury and glial activation in the brain, we acquired per animal images from three sagittal brain sections spaced 100 µm in medial to lateral direction. Image analysis employed two approaches: direct quantitative analysis of fluorescence intensities or deconvolution-supported volumetric estimation of immunoreactive neuropil. For direct quantitative analysis of fluorescence intensities of MAP-2 (cortical layer 3) and GFAP in the frontal cerebral cortex or the hippocampus (CA1), we recorded 2D images of each brain section using a 5 x objective and analyzed the sum of fluorescence intensities (SFLI) values derived from the area of interest. The SFLI values were normalized for the measured area and adjusted for background by subtracting values obtained from sections incubated with secondary antibodies. For analysis by deconvolution microscopy, we recorded 3D images from three fields per brain section with 0.5 µm steps along the Z-axis using a 40 x objective. The Z-stack images were deconvolved using a constrained iterative algorithm and threshold segmentation was applied to estimate the percentage of the neuropil occupied by SYP+ presynaptic terminals. To quantify Iba1+ microglial cells, we counted cell bodies in cerebral cortex (layer 3) or the hippocampus (CA1) on one side of three sagittal sections spaced 100 µm apart medial to lateral. Unless stated otherwise, Slidebook software (version 5 and 6, Intelligent Imaging Innovations, Inc., Denver, CO) was used for all image acquisition and analysis.

Supplementary References

1 Maung, R. *et al.* CCR5 knockout prevents neuronal injury and behavioral impairment induced in a transgenic mouse model by a CXCR4-using HIV-1 glycoprotein 120. *J Immunol* **193**, 1895-1910 (2014).

2 Kaul, M. & Lipton, S. A. Chemokines and activated macrophages in HIV gp120-induced neuronal apoptosis. *Proc Natl Acad Sci U S A* **96**, 8212-8216 (1999).

3 Kaul, M., Ma, Q., Medders, K. E., Desai, M. K. & Lipton, S. A. HIV-1 coreceptors CCR5 and CXCR4 both mediate neuronal cell death but CCR5 paradoxically can also contribute to protection. *Cell Death Differ* **14**, 296-305, doi:10.1038/sj.cdd.4402006 (2007).

4 Medders, K. E., Sejbuk, N. E., Maung, R., Desai, M. K. & Kaul, M. Activation of p38 MAPK is required in monocytic and neuronal cells for HIV glycoprotein 120-induced neurotoxicity. *J Immunol* **185**, 4883-4895 (2010).

5 Sanchez, A. B. *et al.* Antiretrovirals, Methamphetamine, and HIV-1 Envelope Protein gp120 Compromise Neuronal Energy Homeostasis in Association with Various Degrees of Synaptic and Neuritic Damage. *Antimicrob Agents Chemother* **60**, 168-179, doi:10.1128/AAC.01632-15 (2016).

6 Sanchez, A. B. *et al.* CXCL12-induced neurotoxicity critically depends on NMDA receptor-gated and L-type Ca2+ channels upstream of p38 MAPK. *J Neuroinflammation* **13**, 252, doi:10.1186/s12974-016-0724-2 (2016).

7 Hoefer, M. M. *et al.* Combination of methamphetamine and HIV-1 gp120 causes distinct long-term alterations of behavior, gene expression, and injury in the central nervous system. *Exp Neurol* **263**, 221-234 (2015).
